# Comparison of Effectiveness of Supervised Exercise Program and Cyriax Physiotherapy in Patients with Tennis Elbow (Lateral Epicondylitis): A Randomized Clinical Trial

**DOI:** 10.1100/2012/939645

**Published:** 2012-05-02

**Authors:** Rajadurai Viswas, Rejeeshkumar Ramachandran, Payal Korde Anantkumar

**Affiliations:** ^1^BCF College Of Physiotherapy, Indo American Hospital Campus, Kottayam District, Kerala State, Vaikom 686143, India; ^2^Physiotherapist, Rudraksh Physiotherapy Clinic, Kandivali (West), Maharashtra State, Mumbai 400 067, India

## Abstract

*Objective*. To compare the effectiveness of supervised exercise program and Cyriax physiotherapy in the treatment of tennis elbow (lateral epicondylitis). *Design*. Randomized clinical trial. *Setting*. Physiotherapy and rehabilitation centre. *Subjects*. This study was carried out with 20 patients, who had tennis elbow (lateral epicondylitis). *Intervention*. Group A (*n* = 10) had received supervised exercise program. Group B (*n* = 10) was treated with Cyriax physiotherapy. All patients received three treatment sessions per week for four weeks (12 treatment sessions). *Outcome measures*. Pain was evaluated using a visual analogue scale (VAS), and functional status was evaluated by completion of the Tennis Elbow Function Scale (TEFS) which were recorded at base line and at the end of fourth week. *Results*. Both the supervised exercise program and Cyriax physiotherapy were found to be significantly effective in reduction of pain and in the improvement of functional status. The supervised exercise programme resulted in greater improvement in comparison to those who received Cyriax physiotherapy. *Conclusion*. The results of this clinical trial demonstrate that the supervised exercise program may be the first treatment choice for therapist in managing tennis elbow.

## 1. Introduction

The syndrome of persistent disabling pain in the elbow, predominantly in the radio humeral joint, is called as tennis elbow, lateral epicondylitis, or lateral epicondylalgia [1–5]. The definite cause of tennis elbow is not yet known. It is a painful and debilitating musculoskeletal condition that affects health care industry [6]. It is very common in individuals whose jobs necessitate frequent rotary motion of the forearm (e.g., tennis players and carpenters) [7]. It is commonly due to more quick, monotonous, cyclic eccentric contractions and wrist griping activities [8]. The commonly affected arm is the dominant arm, with a prevalence of 1–3% in the general population, but the incidence rapidly increases to 19% between 30–60 years of age and seems to be more severe and long-standing in women [9, 10]. The average period of an episode of lateral epicondylitis ranges between 6 months and 2 years [11]. In tennis elbow, microscopic and macroscopic lesions can be found in the Extensor Carpi Radialis Brevis (ECRB) [12].

The main clinical presentation and the chief complaints in tennis elbow are decreased grip strength, decreased functional activities, and increased pain, which may have significant impact on activities of daily living. The diagnosis of tennis elbow can be made simple, and it may be confirmed by test which would elicit the pain, tenderness over on the facet of the lateral epicondyle on palpation, resisted wrist extension, resisted middle finger extension, and passive wrist flexion [13]. 

Even though tennis elbow has well-defined clinical features, no proper treatment intervention has emanated [14]. In literature, more than 40 different methods have been documented for the treatment of tennis elbow [15]. Conventional treatment [16] for tennis elbow has focused primarily on the pain management by anti-inflammatory medication, ultrasound, phonophoresis [17], or iontophoresis. Various treatments have been attempted for tennis elbow including corticosteroid injection [18], drug therapies, laser [19–22], electrical stimulation [23, 24], ergonomics [25, 26], counterforce bracing [27], acupuncture [28, 29], and splintage [2]. Surgical treatment is indicated in 5–10% [30] of patients who did not improve from their symptoms with conservative treatment approach. The theoretical mechanism of actions of these treatment interventions differs widely, but the entire treatments' goal is to improve function and reduce pain [14]. Even though numerous studies have been conducted on treatment of this clinical condition, till date the most effective management strategy is not agreed [31]. For the treatment of tennis elbow, both medical and physiotherapeutic interventions have been reported in research literature [32]. Cyriax and Cyriax suggested the use of deep transverse friction massage in combination with mill's manipulation for the treatment of tennis elbow [33]. In order to label the treatment intervention as Cyriax physiotherapy, both the treatment components mentioned above must be used jointly in the sequence specified. In this protocol, person must adhere to this intervention 3 times a week for duration of 4 weeks [34]. However, the number of research studies analysing the effectiveness of this treatment intervention is less, the reason being that most of them do not have proper randomization, blinded outcome measures, and accurate functional outcome questionnaires [35–37]. For the above-mentioned reasons, further research is warranted to find out the effectiveness of Cyriax physiotherapy intervention.

The conventional treatment intervention of tennis elbow is most often accompanied by exercise program which may include strengthening, flexibility, or endurance training exercises. For instance, Stasinopoulos et al. [38] recommended the use of static stretching of the Extensor Carpi Radialis Brevis (ECRB) and eccentric strengthening exercises for the wrist extensors in treating lateral epicondylitis. Even though various treatments exist in the management of tennis elbow, optimal treatment intervention is not agreed upon till date. Hence, further research is necessary to find the most effective treatment option in the management of patients with tennis elbow [33]. The purpose of the study was to compare the effectiveness of Cyriax physiotherapy and supervised exercise program in the reduction of pain and improving functional status in patients with tennis elbow.

## 2. Methods

A randomized clinical trial was conducted between March 2011 and September 2011 in an outpatient department, Physiotherapy and Rehabilitation centre, Alleppey, Kerala, India. Patients were referred by orthopaedic consultant, health care providers, and also self-referral to the centre. Patients were included if they were between 30 to 45 years of age and had been diagnosed with tennis elbow, and the duration of symptoms was between 8 and 10 weeks.

### 2.1. Inclusion Criteria

1. Pain with gripping.
2. Pain with resisted wrist extension.
3. Pain with passive wrist flexion with the elbow extension.
4. Tenderness on palpation over the lateral epicondyle of humerus.

### 2.2. Exclusion Criteria

1. Cardiovascular diseases.
2. Neurological impairments.
3. Aversion to manual contact.
4. Neuromuscular diseases.
5. Previous trauma to the elbow region.
6. Elbow pain.
7. Previous surgery to the elbow region.
8. Peripheral nerve entrapment.
9. Cervical radiculopathy.
10. Corticosteroid injection within 6 months.
11. Previous therapy for elbow joint (minimizing expectation bias).

All patients signed the written consent form prior to participation. The recruited patients had also completed a standard health questionnaire which encompassed details relating to patient demographics, duration of symptoms, any previous treatment undertaken, and job status.

### 2.3. Treatment

Patients assigned to Group A received supervised therapeutic exercise program which included static stretching of the Extensor Carpi Radialis Brevis followed by eccentric strengthening of the wrist extensors. Static stretching was performed in the seated position with elbow extension, forearm pronation, and wrist flexion with ulnar deviation. According to the patient tolerance stretch force was applied. This stretch position was held for duration of 30–45 seconds and was performed 3 times before and 3 times after the eccentric exercise portion of the treatment for a total of 6 repetitions [14]. There was a 30-second rest interval between each bouts of stretching.

Eccentric strengthening exercise was performed in the seated position with full elbow extension, forearm pronation, and maximum wrist extension. From this position, the patient slowly lowered wrist into flexion for a count of 30, using the contralateral hand to return the wrist to maximum extension. Patients were instructed to continue the exercise even when they experience mild discomfort and to stop the exercise if the pain worsens and becomes disabling. For whom the eccentric exercise could be performed without minor discomfort or pain, the load was increased using free weights based on the patients 10 RM (Repetition Maximum). Three sets of ten repetitions were performed during each treatment, with a one-minute rest interval between each set. Patients were also provided with education manual regarding ergonomics and activity modification technique to avoid aggravation of symptoms.

**Figure 1:**
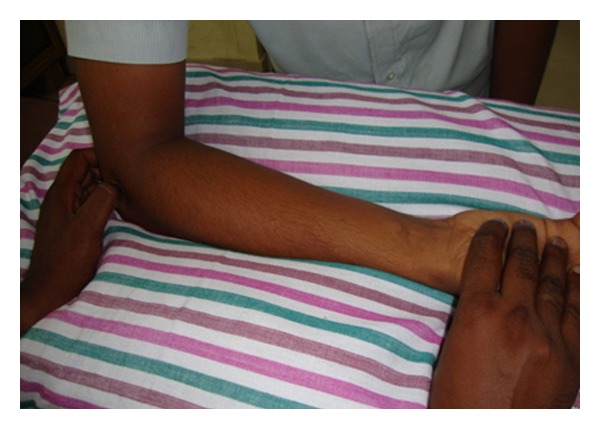
Deep transverse friction massage.

Patients in Group B received Cyriax physiotherapy, which consists of 10 minutes of deep transverse friction massage immediately followed by a single application of Mill's manipulation. The hand placement is shown in Figure 1. Deep transverse friction for tennis elbow is applied as follows [34, 39]. The patient should be positioned comfortably with the elbow fully supinated and in 90° of flexion. After palpating the anterolateral aspect of the lateral epicondyle of humerus, the area of tenderness was mapped. Deep transverse friction is applied with the side of the thumb tip. The pressure was applied in a posterior direction on the tenoosseous junction. It was applied for ten minutes after the numbing effect has been attained, to prepare the tendon for Mill's manipulation [33].

**Figure 2:**
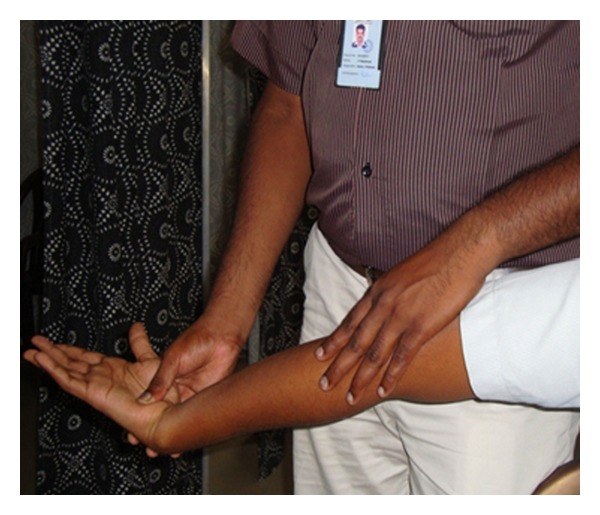
Mill's Manipulation.

For the technique of Mills manipulation, patients were positioned comfortably in the seating position with the affected extremity in 90° of abduction with internal rotation enough so that the olecranon faced up. The therapist stabilized the patient's wrist in full flexion and pronation with one hand, while other hand was placed over the olecranon [14]. While assuming full wrist flexion and pronation position, the therapist should apply a high-velocity low-amplitude thrust at the end range of elbow extension (Figure 2).

### 2.4. Outcome Measures

Outcome measures used in the study includes pain intensity and functional status which were recorded at base line (pretest) and at the end of 4 weeks. An independent observer, who was blinded to the patient group allocation, assessed the outcome measures. Pain intensity was measured using the visual analogue scale (VAS). The VAS consists of a 10 cm horizontal line with two ends labelled as 0 cm representing the “least pain imaginable” and 10 cm the “worst pain imaginable”. Patients were given instructions to intersect this VAS scale with a vertical line depending on their current level of pain. The VAS assessment tool has been found to be a valid and also a reliable method of measuring patients perceived pain [40, 41].

Patients functional status was assessed by completion of the Tennis Elbow Function Scale (TEFS) [42]. In TEFS scale, the patients were instructed to perform certain set of task that can be difficult in performing as a result of their problem and were informed to accordingly rate the intensity of their pain. Higher scores are indicative of greater levels of disability. The TEFS assessment tool has been found to have high test-retest reliability (ICC 0.92) and moderate construct validity (Pearson's correlation coefficient 0.47) [42].

## 3. Data Analysis

**Figure 3:**
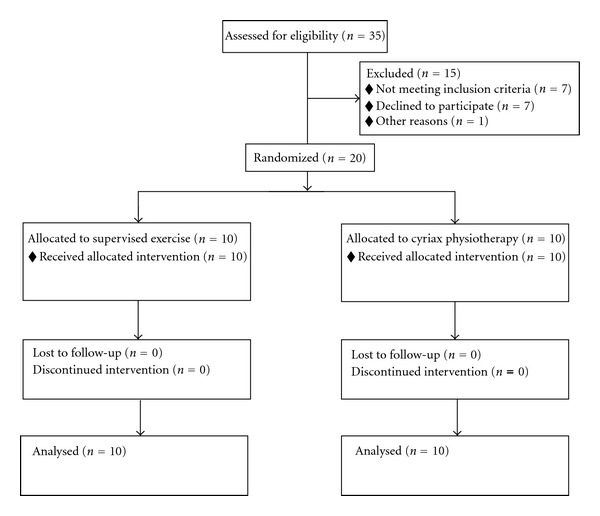
Participants flow chart.

Thirty-five patients, 20 male and 15 female, were initially assessed for eligibility for this study. 15 patients were excluded for the following reasons: not meeting inclusion criteria (*n* = 7), declined to participate (*n* = 7), and other reason (*n* = 1). The remaining 20 patients (10 males and 10 females) randomly allocated into 2 groups. Participant flow through the study is illustrated in Figure 3. Patients in Group A received supervised exercise program while patients in Group B received Cyriax Physiotherapy treatment. All patients were seen 3 times a week for 4 weeks for a total of 12 treatment sessions.

Data analysis was performed with SPSS version 16.0. Statistical analysis including mean and standard duration was calculated for all measurement. The mean differences with standard deviation for outcome measures of pain intensity and function scale were calculated before the treatment and also the end of 4 weeks. Mann Whitney *U* test, Wilcoxon Signed Rank test, and two sample *t*-test are the statistical tests used in this study.

## 4. Results

At the time of initial evaluation, statistical analysis did not reveal any significant differences for any of the variables between Group A (supervised exercise program) and Group B (Cyriax physiotherapy).

### 4.1. Age Distribution

**Figure 4:**
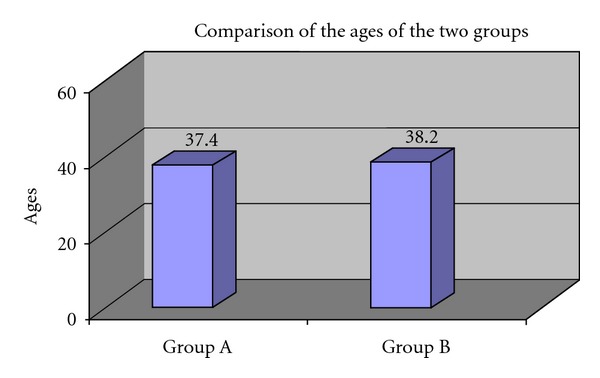
Comparison of the ages of the two groups.

Statistical tool used is the two sample *t* test. For Group A, the age of the subjects ranged between 30 and 45, while for Group B it ranged between 31 and 45. The mean age for Group A was 37.40 ± 4.881 and Group B was 38.20 ± 4.341 as shown in Figure 4 and Table 1. The intergroup comparison of mean age did not show any significant difference between the ages of the two groups.

### 4.2. Gender Distribution

**Figure 5:**
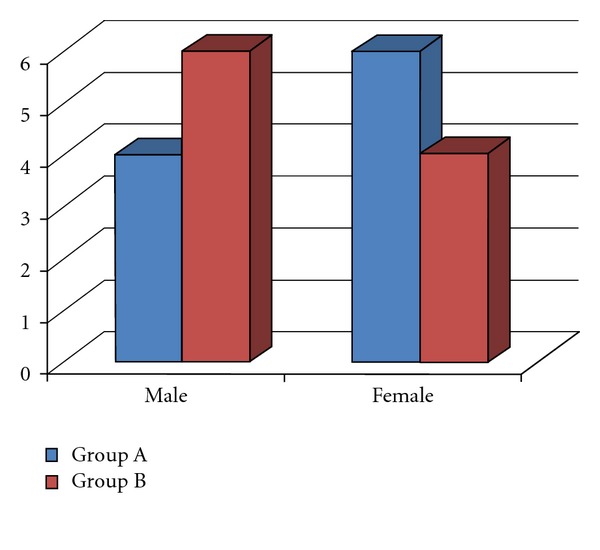
Distribution of gender in both groups.

Group A consisted of 10 patients (*n* = 10), with a gender distribution of 4 males (40%) and 6 females (60%). Group B also consisted of 10 patients (*n* = 10) and a gender distribution of 6 males (60%) and 4 females (40%). These data were presented in Figure 5 and Table 2.

### 4.3. Duration of Symptoms

**Figure 6:**
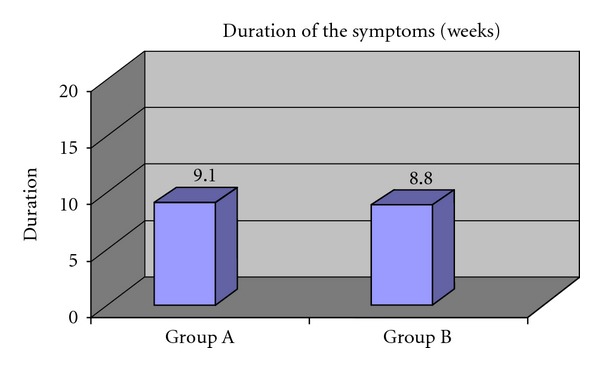
Duration of symptoms (in weeks) in both groups.

The mean duration of symptoms (in weeks) for Group A was 9.1 ± 0.88 and for Group B was 8.8 ± 0.91 weeks. There is no significant difference between the duration of symptom of the two groups at 5% level of significance as shown in Figure 6 and Table 3.

### 4.4. Visual Analogue Scale (VAS)

VAS scores were found to be similar between groups at baseline (Pretest). Statistical tool used is the Mann-Whitney *U* test. There is no significant difference between the pre-VAS scores of the two groups at 5% level of significance (Table 4).

#### 4.4.1. Tennis Elbow Function Scale (TEFS)

TEFS scores were found to be similar between groups at baseline (Pre Test). Statistical tool used is the Mann-Whitney *U* test. There is no significant difference between the pre-TEFS scores of the two groups at 5% level of significance (Table 5).

#### 4.4.2. Pre-Post Test Comparison of VAS Scores in Group A

The intragroup comparison of pain intensity as measured by VAS at the end of treatment intervention in Group A, presented in Tables 6(a) and 6(b), shows that there was a definitive reduction in the pain intensity at the end of 4 weeks of supervised exercise program. The statistical test used is Wilcoxon signed-rank test.

#### 4.4.3. Pre-Post Test Comparison of VAS Scores in Group B

The intragroup comparison of pain intensity as measured by VAS at the end of treatment intervention in Group B, presented in Tables 7(a) and 7(b), shows that there was a definitive reduction in the pain intensity at the end of 4 weeks of Cyriax physiotherapy treatment. The statistical test used is Wilcoxon signed-rank test.

#### 4.4.4. Posttest Comparison of VAS Scores between the Groups

**Figure 7:**
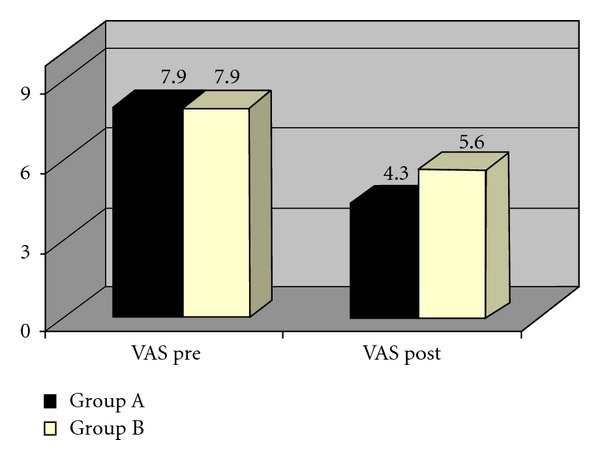
Comparison of VAS scores of two groups.

The results of the posttest intergroup comparison of pain intensity as measured by VAS are presented in Tables 8(a) and 8(b). Though both groups showed significant reduction in pain when compared to the pretest score, the intergroup comparison of VAS scores showed a higher reduction in VAS scores in Group A than Group B, which was statistically significant. The statistical tool used is Mann-Whitney *U* test (Figure 7).

#### 4.4.5. Pre-Post Test Comparison of TEFS Scores in Group A

The intragroup comparison of functional status as measured by TEFS at the end of treatment intervention in Group A, presented in Tables 9(a) and 9(b), shows that there was a definitive improvement in the functional status at the end of 4 weeks of supervised exercise program. The statistical test used is Wilcoxon signed-rank test.

#### 4.4.6. Pre-Post Test Comparison of TEFS Scores in Group B

The intragroup comparison of functional status as measured by TEFS at the end of treatment intervention in Group B, presented in Tables 10(a) and 10(b), shows that there was a definitive improvement in the functional status at the end of 4 weeks of Cyriax physiotherapy treatment. The statistical test used is Wilcoxon signed-rank test.

#### 4.4.7. Posttest Comparison of TEFS Scores between the Groups

**Figure 8:**
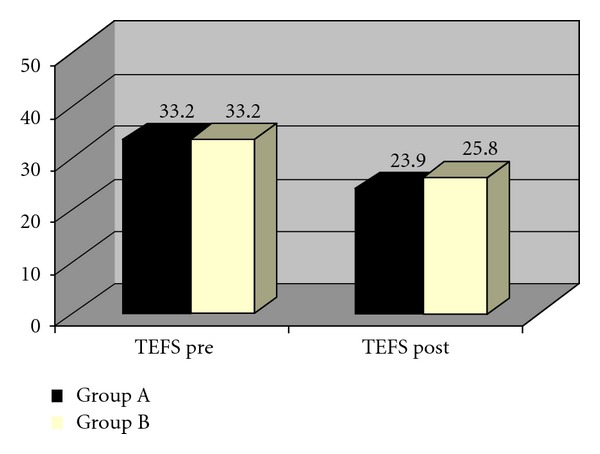
Comparison of TEFS scores of two groups.

The results of the posttest intergroup comparison of functional status as measured by TEFS are presented in Tables 11(a) and 11(b). Though both Groups showed significant improvement in the functional status when compared to the pretest score, the intergroup comparison of TEFS scores showed a higher reduction in TEFS scores in Group A than Group B, which was statistically significant. The statistical tool used is Mann-Whitney U test (Figure 8).

## 5. Discussion

The results of this study demonstrate that both the supervised exercise program (Group A) and Cyriax physiotherapy treatment (Group B) groups experienced significant improvements in pain and function following 4 weeks treatment sessions. The supervised exercise and static stretching group experienced greater outcomes for all variables in comparison to those receiving Cyriax physiotherapy treatment. The reported success of supervised exercise program in this study is consistent with previously published research studies [12, 36, 37]. Pienimäki et al. compared a six-to-eight-week exercise programme of stretches and exercises (isometric and isotonic) with a treatment of pulsed ultrasound across the same time span and showed that the SMD for pain visual analogue scale at rest was 0.97 (95% CI 0.30 to 1.63) and 0.66 (95% CI 0.01 to 1.31) for pain visual analogue scale under strain. Maximum grip strength was not significantly different between groups [12]. This suggests a favourable effect in that exercise may improve pain in lateral epicondylalgia but not maximum grip strength [12]. Verhaar et al. compared the effects of corticosteroid injections with Cyriax physiotherapy in treating patients with tennis elbow. The results showed that the corticosteroid injection was significantly more effective on the outcome measures (pain, function, grip strength, and global assessment) than Cyriax physiotherapy at the end of the treatment, but at the followup, one year after the end of treatment, there were no significant differences between the two treatment groups [37]. Stasinopoulus et al. compared the effectiveness of supervised exercise, Cyriax physiotherapy, and treatment with polychromatic noncoherent light in managing tennis elbow. They concluded that supervised exercise consisting of static stretching and eccentric strengthening produced the largest effect in reducing pain and improving function [36].

The early return of functional status is very useful for a sports person, as it will facilitate his/her return to sports in less duration. This improvement in functional status will also prevent disuse atrophy or muscle weakness resulting from less or no activity due to pain and disability caused by tennis elbow. It has been assumed that the underlying mechanism of pain relief secondary to friction massage may be due to modulation of pain impulses at the spinal cord level [43]. At present, no published evidence exists to support the proposed mechanism as to what actually occurs during and following manual treatment with Cyriax physiotherapy [33]. The hypothesized mechanism of Mill's manipulation is the lengthening of scar tissue following the rupture of adhesions due to the manipulation [33]. In comparing the results of these trials to those experienced by the supervised exercise treatment group in the present study, two points must be considered. First, none of the above-mentioned trials used a true control group, thereby not controlling for the natural course of the disorder or spontaneous recovery. Second, the present study did not assign patients to receive supervised exercise as an isolated treatment. Therefore, comparisons between our results and those of previous trials should be made with caution as it is not possible to determine which intervention made the greatest contribution to the treatment effect.

## 6. Limitations of This Study

- No follow-up data was collected; therefore, the long-term effects of the interventions in the present study remain unknown.
- Absence of true control group affects the internal validity of the study.

## 7. Conclusions

We rejected the null hypothesis that no difference would be seen in pain intensity and functional status after 4 weeks as compared with Cyriax physiotherapy treatment. The groups that performed supervised exercise program for 4 weeks showed significantly greater improvement in reduction of pain and functional status than the Cyriax physiotherapy treatment. The favorable results in the present study indicate the need for future research examining the incorporation of supervised exercise program into multimodal treatment regimens.

## Figures and Tables

**Table 1:** Mean, standard deviation (SD), & standard error (SE) of age.

Age comparison	*n*	Mean	SD	Standard error mean	*P* value	Result
Group A	10	37.40	4.88	1.543	1.000	*P* > 0.05 (not significant)
Group B	10	38.20	4.34	1.373		

**Table 2:** Percentage of distribution of gender in both groups.

	Male	Female
Group A	4 (40%)	6 (60%)
Group B	6 (60%)	4 (40%)

**Table 3:** Duration of symptoms (in weeks) in both groups.

Duration of symptoms in weeks	Mean	SD	*P* value	Result
Group A	9.1	0.88	1.000	*P* > 0.05 (Not Significant)
Group B	8.8	0.91		

**Table:** (a) Ranks

	Group	*n*	Mean rank	Sum of ranks
VAS Pre	Group A	10	10.50	105.00
	Group B	10	10.50	105.00

	Total	20		

**Table:** (b) Test statistics

	VAS Pre
Mann-Whitney *U*	50.000
Exact significance	1.000

**Table:** (a) Ranks

	Group	*n*	Mean rank	Sum of ranks
TEFS Pre	Group A	10	10.50	105.00
	Group B	10	10.50	105.00

	Total	20		

**Table:** (b) Test statistics

	TEFS Pre
Mann-Whitney *U*	50.000
Exact significance	1.000

**Table:** (a) Ranks

		*n*	Mean rank	Sum of ranks
VAS post- VAS pre	Negative ranks	10	5.50	55.00
	Positive ranks	0	.00	.00
	Ties	0		

	Total	10		

**Table:** (b) Test statistical

	VAS post- VAS pre
Z	−2.889
Asymp. significance ( 2-tailed)	.004

**Table:** (a) Ranks

		*N*	Mean rank	Sum of ranks
VAS post- VAS pre	Negative ranks	10	5.50	55.00
	Positive ranks	0	.00	.00
	Ties	0		

	Total	10		

**Table:** (b) Test statistics

	VAS post- VAS pre
Z	−2.919
Asymp. significance	.004

**Table:** (a) Ranks

	Group	*n*	Mean rank	Sum of ranks
VAS Post	Group A	10	7.10	71.00
	Group B	10	13.90	139.00

	Total	20		

**Table:** (b) Test statistics

	VAS Post
Mann-Whitney *U*	16.000
Exact significance	.009

**Table:** (a) Ranks

		*n*	Mean rank	Sum of ranks
TEFS Post-TEFS Pre	Negative ranks	10	5.50	55.00
	Positive ranks	0	.00	.00
	Ties	0		

	Total	10		

**Table:** (b) Test statistics

	TEFS Post-TEFS Pre
Z	−2.859
Asymp. significance	.004

**Table:** (a) Ranks

		*n*	Mean rank	Sum of ranks
TEFS Post-TEFS Pre	Negative ranks	10	5.50	55.00
	Positive ranks	0	.00	.00
	Ties	0		

	Total	10		

**Table:** (b) Test statistics

	TEFS Post-TEFS Pre
Z	−2.889
Asymp. significance	.004

**Table:** (a) Ranks

	Group	*n*	Mean rank	Sum of ranks
TEFS Post	Group A	10	6.65	66.50
	Group B	10	14.35	143.50

	Total	20		

**Table:** (b) Test statistics

	VAS Post
Mann-Whitney *U*	11.500
Exact significance	.002
